# The Historical Medical Museum

**Published:** 1913-07-05

**Authors:** 


### THE HISTORICAL MEDICAL MUSEUM.

As has been already noted in these columns, the Historical Medical Museum which Mr. H. S. Wellcome has been preparing for a long time past has now come into being; and a very wonderful place it is. With his own extensive collections, and the assistance of a host of exhibitors of articles on loan, Mr. Wellcome has produced an exhibition of interesting objects bearing on every aspect of ancient and mediæval and modern medicine. To mention even a fractional percentage of the treasures now housed at 54a Wigmore Street would involve a small catalogue in itself.

Many of the exhibits have, as is natural, an interest mainly medical; others appeal to all sorts and conditions of men; and others, again, have an especial institutional significance. First and foremost among the latter may be mentioned the (reconstruction of an) Italian hospital ward of the sixteenth century. The small room shown has no windows, though whether this is intended to represent faithfully an original remains in doubt. Apart from this, the scene shown might almost stand for the twentieth century instead of the sixteenth; for there are still plenty of hospitals in Italy, Spain, and other Mediterranean countries in which no higher standard of hospital construction and equipment has yet been reached. The sixteenth-century lying-in room is another interesting reconstruction of distinctly realistic conception. The figures of the baby and its attendant are, perhaps, the most carefully carried out.

A fifteenth-century alchemist's laboratory, fully equipped with original apparatus and appliances of the period and with models of the actual furnaces used by those mediæval magicians, is perhaps of more general than particularly institutional interest; but it is a most fascinating exhibit, complete even to the stuffed crocodile which was an indispensable adjunct of any high-class alchemist's establishment. The hospital pharmacy of the sixteenth century is chiefly noticeable for the splendid collection of pottery which it contains. Another fine exhibit of this type is the eighteenth-century London pharmacy, containing the actual original shop-front of the first Bell, founder of the Pharmaceutical Society, and of the business which, after amalgamations and removals, is now carried on in Wigmore Street a few yards from Mr. Wellcome's museum. The vases and ewers of this shop are of Davenport ware, the ointment jars of Staffordshire stoneware, and the essence bottles of red Venetian glass. Connoisseurs of these branches of art will find this shop well worth a visit.

The relics of various heroes of bygone days are interesting, not merely by reason of their illustrious owners, but because they also serve to show the state of contemporary medical and surgical progress. Thus Nelson's medicine-chest on the *Victory*, Livingstone's pocket instrument-case, Wellington's Peninsular War medicine-chest, and similar exhibits are certain to be sought out by visitors.

The paintings and prints are very numerous, and many of them represent persons prominent in the institutional world in their day. Probably the representations of Miss Nightingale will attract most attention from hospital workers of the present day; though Lord Lister's portraits may run them close. Of hospital worthies of more remote times there is a plentiful supply, including John Hunter, Abernethy, Brodie, Cooper, Jenner, and many others. Compared with the pictures of old-time medical practice and methods, there are not very many pictures of mediæval hospitals. One represents a scene at a hospital for incurables; another a surgical operation in a seventeenth-century hospital ward; and there are others. But, broadly speaking, this part of the exhibition does not attain a high level of purely institutional interest. Finally, it is a great pleasure to acknowledge the courtesy with which our representative was received and shown round this unique and extremely interesting collection.

